# Clustering of multiple health risk behaviors and its association with diabetes in a Southern Chinese adult population: a cross-sectional study

**DOI:** 10.7717/peerj.9025

**Published:** 2020-05-11

**Authors:** Guanrong Zhang, Caibing Luo, Ying Cui, Yifan Lu, Yang Yang

**Affiliations:** 1 Information and Statistics Center, Guangdong Provincial People’s Hospital, Guangdong Academy of Medical Sciences, Guangzhou, Guangdong, China; 2 State Key Laboratory of Oncology in South China, Logistics Department, Sun Yat-sen University Cancer Center, Guangzhou, Guangdong, China; 3 Guangdong Eye Institute, Department of Ophthalmology, Guangdong Provincial People’s Hospital, Guangdong Academy of Medical Sciences, Guangzhou, Guangdong, China; 4 Harvard Medical School, Boston, MA, United States of America

**Keywords:** Health risk behavior, Clustering, Latent class analysis, Diabetes

## Abstract

**Background:**

Identifying the clustering patterns of health risk behaviors (HRBs) within individuals and their health impacts are essential to develop lifestyle promotion strategies. This study aimed to explore the clustering of a range of HRBs and the associations between such identified clusters and diabetes in Southern Chinese adults.

**Methods:**

Data from 5,734 adults aged 35–75 years and underwent health examinations from November 2012 to December 2013 at a tertiary hospital in Guangzhou were analyzed. Behavioral characteristics, including smoking, alcohol use, physical activity, and sleep duration and quality, were measured by questionnaires. Latent class analysis was conducted by gender to identify HRBs clustering patterns, and logistic regression models were used to estimate the associations between behavioral patterns and diabetes.

**Results:**

Three distinct behavioral clusters emerged in both genders. Male classes were defined as: (1) healthy lifestyle (Class 1, 62.9%); (2) cumulate harmful habits (Class 2, 27.1%); (3) poor sleep and risky habits (Class 3, 10.0%). Female classes were: (1) healthy lifestyle (Class 1, 83.0%); (2) inactive, daytime dysfunction (Class 2, 5.7%); (3) poor sleep habits (Class 3, 11.3%). Individuals of Class 2 and Class 3 showed a higher likelihood of diabetes across genders (multivariable-adjusted ORs [95% CIs], 2.03 [1.49–2.76] and 2.61 [1.78–3.81] among males, 2.64 [1.16–5.98] and 1.81 [1.07–3.06] among females) when compared with those of Class 1.

**Conclusions:**

Our data provided additional evidence of HRBs clustering among adults, and such clustering was associated with an increased risk of diabetes. These findings have implications for identifying vulnerable subgroups and developing diabetes prevention programs.

## Introduction

Health risk behaviors (HRBs) have been increasingly recognized as the main cause of chronic diseases, including cardiovascular disease (CVD), some cancers, and diabetes (GBD 2017 Risk Factor Collaborators, 2018; Islami et al., 2018). A large body of evidence suggested that HRBs, including cigarette smoking, heavy alcohol consumption, physical inactivity, and suboptimal diet, are among the most common and preventable causes of non-communicable diseases and death worldwide (Department of Health & Human Services, 2014; GBD 2017 Diet Collaborators, 2019; Lee et al., 2012; Rehm et al., 2017). Additionally, sleep disturbances such as short sleep duration and poor sleep quality have emerged to being linked to an increased risk of adverse cardiometabolic outcomes and all-cause mortality (St-Onge et al., 2016), which is comparable to that of traditional risk factors like physical inactivity. Therefore, promoting changes in HRBs is of highest importance for public health.

There is evidence that HRBs usually co-occur as clusters, which means that the co-occurrence of multiple risk behaviors in individuals is more prevalent than expected by chance (Noble et al., 2015). Data from many countries showed that over 50% of adults partake in two or more risk behaviors (John, Hanke & Freyer-Adam, 2018; Li et al., 2012; Watts et al., 2016); and people with such characteristics often present a dramatically increased risk for future serious health issues (Krokstad et al., 2017; Li et al., 2018), suggesting a synergistic effect among HRBs.

A growing number of studies have examined the clustering of HRBs in adults, and most of them focused on several conventional determined risk behaviors like smoking, alcohol use, physical inactivity, and unhealthy diet (Meader et al., 2016; Noble et al., 2015). However, because of the differences in analytical approaches and the range of HRBs, there remains little consensus about what behaviors were most likely to cluster. Furthermore, although detailed information about the impact of sleep as a novel risk factor on chronic disease or mortality is available, few studies have investigated the clustering between sleep and other established HRBs, as well as their combined effects on health outcomes. Two recent studies from developed countries have found distinct risk clusters of traditional and emerging behavioral factors, which are strongly associated with all-cause mortality and obesity (Ding et al., 2015; Perez-Rodrigo et al., 2017). Nevertheless, there are disparities in lifestyle behaviors among different sociodemographic populations (Meader et al., 2016; Pampel, Krueger & Denney, 2010). In view of this, deepening insight into the clustering patterns of HRBs in Chinese adults is crucial to design and implement health promoting policies.

Along with ageing population and rapid changes in lifestyle, diabetes has reached epidemic proportions in China during recent decades (Xu et al., 2013). Many studies have indicated the joint effects of the co-occurrence of lifestyle behaviors on diabetes (Long et al., 2015; Shan et al., 2018), including tobacco and alcohol use, physical activity, and sleep. However, most of these studies focused on predefined patterns or counting number of healthy/unhealthy behaviors. Such approaches may probably provide only a partial picture of concurrent behaviors as well as their influences on diabetes. Instead, some recent studies incorporated data-driven analytic techniques (e.g., cluster analysis, latent class analysis (LCA), etc.) to explore risk factor clusters related to diabetes. For instance, Wang et al. (2017) identified four different subgroups based on lifestyle behaviors among patients with type 2 diabetes. They reported that an unhealthy diet and less activity lifestyle is associated with poor diabetes-related outcomes. Another study investigated the clustering of complications and comorbidities in a sample of Chinese diabetic patients (Gao et al., 2017). According to this study, unhealthy behaviors are correlated with a higher prevalence of complications and comorbidities. Despite these associations, there is limited research examining the link between patterns of behavior combinations and diabetes among the general population in China. Identification of subgroups based on HRBs in adults can help to detect the high-risk populations and develop targeted strategies for diabetes prevention. Therefore, the aims of this study are: (1) to identify meaningful clustering patterns of lifestyle behaviors among adults in Guangzhou, Southern China; (2) to evaluate the associations between different lifestyle clusters and diabetes.

## Methods

### Study population

This study was conducted retrospectively. Data were gathered from the health record database for employees, who participated in the annual health examination at the Health Management Center of a tertiary hospital in Guangzhou. To obtain baseline data for health management, adults aged 35–75 years from 64 institutions were invited to complete an additional questionnaire survey from November 2012 to December 2013. The questionnaire was self-administered and included questions on demographics, lifestyle behaviors, personal and family history of CVD and diabetes. Verbal consent was obtained from participants before answering. All subjects involved in the survey were screened for eligibility and those with missing data in relevant variables were excluded. This study protocol was approved by the Research Ethics Committee of Guangdong Provincial People’s Hospital (10 December 2018; approval No. GDREC2018483H).

### Measurements

#### Behaviors variables

Five behavioral characteristics, including smoking, alcohol use, physical activity, and nocturnal sleep duration and quality, were collected from the written questionnaire. Tobacco use was assessed via a question about the average number of daily cigarettes smoking. Current smoking referred to at least one cigarette per day within the last 6 months, consistent with the definition of the 2010 national smoking survey of Chinese adults (Liu et al., 2017). Alcohol use was derived from responses to two questions on the frequency (days per week) and amount (drinks per day) of alcohol consumption in the past 12 months. One drink was defined as 14 g of pure alcohol, which equates to a 150 ml glass of wine, a 350 ml can of beer, or two shots of spirits. We multiplied the drinking frequency by quantity consumed per drinking day to compute the weekly consumption of alcohol. As in previous literatures from other investigators (Xi et al., 2017; Zhu et al., 2019), we defined heavy drinking as consuming more than 14 drinks per week for men (7 for women). Physical activity was estimated based on recall of a typical week, by asking the frequency and time spent on leisure-time exercise, such as walking, dancing, jogging or playing sports. Consistent with guidelines for Chinese adults (Bureau for Disease Control and Prevention, Ministry of Health, People’s Republic of China, 2011), the two responses were combined and categorized into three levels: active (≥150 min/week of moderate or vigorous activity), moderately active (exercise but not at active level), and inactive (no exercise). Habitual sleep duration was measured as self-reported average hours of sleep per night during the passing year, with categories of <6, 6∼, 7∼, and ≥8 h. Sleep quality was assessed using the Athens Insomnia Scale (AIS), which has been ascertained as a reliable and valid tool for screening of insomnia (Chung, Kan & Yeung, 2011). The AIS consists 8 items: the first 5 items refer to the difficulty with sleep induction, awakening during the night, final awakening earlier than desired, total sleep duration, and overall quality of sleep, while another 3 items pertain to the sense of well-being, functioning and sleepiness during the daytime on the following day. Participants were requested to rate each item from 0 to 3 (with 0 corresponding to no problem at all and 3 to a very serious problem) based on the symptoms of sleep difficulty during the previous month. Since few responders reported serious problems, data were transformed into dichotomous variables, where scores 0 and 1 were combined as “insignificant difficulty”, and scores 2 and 3 were combined as “significant difficulty”.

#### Physiological measures and definitions

Data on anthropometric (body weight, height and blood pressure [BP]) and fasting plasma glucose (FPG) were gathered from the health examination recodes using a standardized electronic format. All physical measurements were conducted following a standard operation procedure by trained physicians, and biochemical variables were assessed at the central laboratory of a tertiary hospital. Data from 5% of studies were randomly selected for quality and consistency review by two investigators independently. Body mass index (BMI) was calculated as weight (kg) divided by height squared (m^2^). Diabetes was defined as FPG ≥7.0 mmol/L, or current use of anti-diabetic medications, or a history of physician-diagnosed diabetes.

#### Covariates

Demographic (age, education level, family history of diabetes) and clinical variables (BMI, BP) were considered as potential confounders when examining the relationship of HRBs with diabetes based on previous literature.

### Statistical analysis

All analyses were stratified by gender, considering the known differences in lifestyle behaviors between males and females. Numerical variables with normal distribution were expressed as mean (standard deviation, SD) and compared using one-way analysis of variance; while those with skewed distribution were expressed as median (interquartile range, IQR) and analyzed with nonparametric methods. Categorical data were presented as frequency (percentage) and were compared using *χ*^2^ test.

To assess clustering patterns of HRBs among the participants, LCA was performed. Unlike traditional clustering techniques that focus on relations among variables, LCA is a person-centered analytic approach in which individuals are classified into homogenous subgroups based on similar characteristics (Miettunen et al., 2016). In our study, smoking, alcohol use, physical activity status, sleep duration, and eight symptoms of insomnia, were treated as behavioral indicators. In addition, age and education level were added to the analysis as covariates. To identify the optimal number of clusters, models with two to five classes were fitted. Several fit statistics including Akaike information criterion (AIC), Bayesian information criterion (BIC), adjusted BIC (aBIC), entropy, and the Lo-Mendell-Rubin likelihood ratio test (LMR) were employed for model comparison. The final model was selected based upon the IC indices (lower values indicate better fit), combining with the substantive meaning of estimated clusters. Individuals were assigned to the most probable class according to their posterior probabilities of belonging to each cluster. To test for measurement invariance across genders, multiple group LCA was conducted by comparing the model with free measurement parameter estimation with another model where measurement paraments were restricted across gender. The difference in fit statistics between these models was statistically significant (*χ*^2^_restricted_- *χ*^2^_unrestricted_ = 623.82, *df*_restricted_-*df*_unrestricted_ = 45, *p* < 0.001), implying that measurement invariance across genders did not hold. Thus, separate models for each gender should be estimated.

The associations between HRBs clusters and diabetes were analyzed using the logistic regression models. All models were adjusted for the covariates, and results were presented as odds ratios (ORs) with 95% confidence intervals (CIs). Finally, we conducted sensitivity analyses to evaluate potential reverse causality by excluding patients with self-reported history of cardiometabolic diseases (hypertension, coronary heart disease, stroke, and diabetes). Analyses were carried out in Mplus 7.1 (Muthén & Muthén, Los Angeles, CA, USA) and SPSS 24.0 (SPSS Inc., Chicago, IL, USA). The statistical significance level was 0.05 (two-tailed).

## Results

### Sample characteristics

A total of 6,551 participants were identified from the electronic health records over the study period. Of these, 817 (12.5%) were excluded due to missing values on alcohol consumption, physical activity, FPG, weight, height, or BP. Thus, the final analysis included 5,734 subjects (3,593 males and 2,141 females).

Demographic, behavioral and clinical characteristics of the study sample were shown in Table 1. The mean age was 47.6 years for men and 48.3 years for women. Over 75% of the individuals failed to meet the recommended weekly amount of 150 min of moderate to vigorous activity, and almost 50% slept for <7 hours/night in both genders. In addition, men also had a high prevalence of smoking and heavy alcohol drinking. Diabetes was present in 354 (6.2%, 95% CI [5.6–6.8]%) participants, including 251 (7.0%, 95% CI [6.2–7.8]%) males and 103 (4.8%, 95% CI [3.9–4.7]%) females.

**Table 1:** Demographic, health risk behavior and clinical characteristics of the analytical sample stratified by gender.

	Males (*n*= 3,593)		Females (*n*= 2,141)		Overall (*n*= 5,734)	
Characteristics	*n*	%/Mean ± SD	*n*	%/Mean ± SD	*n*	%/Mean ± SD
Age (years)		47.6 ± 9.5		48.3 ± 10.0		47.8 ± 9.7
Education (College and above)	2,164	60.2	1,256	58.7	3,420	59.6
Family history of diabetes	676	18.8	558	26.1	1,234	21.5
Current smoking	1,476	41.1	10	0.5	1,486	25.9
Heavy alcohol drinking	925	25.7	60	2.8	985	17.2
Physical activity						
Active	834	23.2	517	24.1	1,351	23.6
Moderately active	1,502	41.8	701	32.7	2,203	38.4
Inactive	1,257	35.0	923	43.1	2,180	38.0
Nocturnal sleep duration						
<6 h	530	14.8	337	15.7	867	15.1
6∼h	1,286	35.8	638	29.8	1,924	33.6
7∼h	1,326	36.9	811	37.9	2,137	37.3
≥8 h	451	12.6	355	16.6	806	14.1
Items of Athens insomnia index						
Difficulty with sleep induction	230	6.4	272	12.7	502	8.8
Awakening during the night	224	6.2	216	10.1	440	7.7
Final awakening earlier than desired	196	5.5	180	8.4	376	6.6
Insufficient total sleep duration	336	9.4	285	13.3	621	10.8
Unsatisfactory overall quality of sleep	306	8.5	288	13.5	594	10.4
Decreased sense of well-being during the day	62	1.7	63	2.9	125	2.2
Decreased functioning during the day	164	4.6	140	6.5	304	5.3
Sleepiness during the day	537	14.9	278	13.0	815	14.2
Body mass index (kg/m^2^)		24.4 ± 2.8		22.6 ± 2.9		23.7 ± 3.0
Systolic blood pressure (mmHg)		131.1 ± 16.2		127.1 ± 19.6		129.6 ± 17.6
Diastolic blood pressure (mmHg)		79.0 ± 10.9		74.7 ± 10.6		77.4 ± 11.0
Fasting plasma glucose (mmol/L)		5.2 ± 1.3		5.0 ± 1.1		5.1 ± 1.3
Diabetes	251	7.0	103	4.8	354	6.2

**Notes.**

SD standard deviation *n* frequency % proportion

### LCA and class patterns

**Figure 1:**
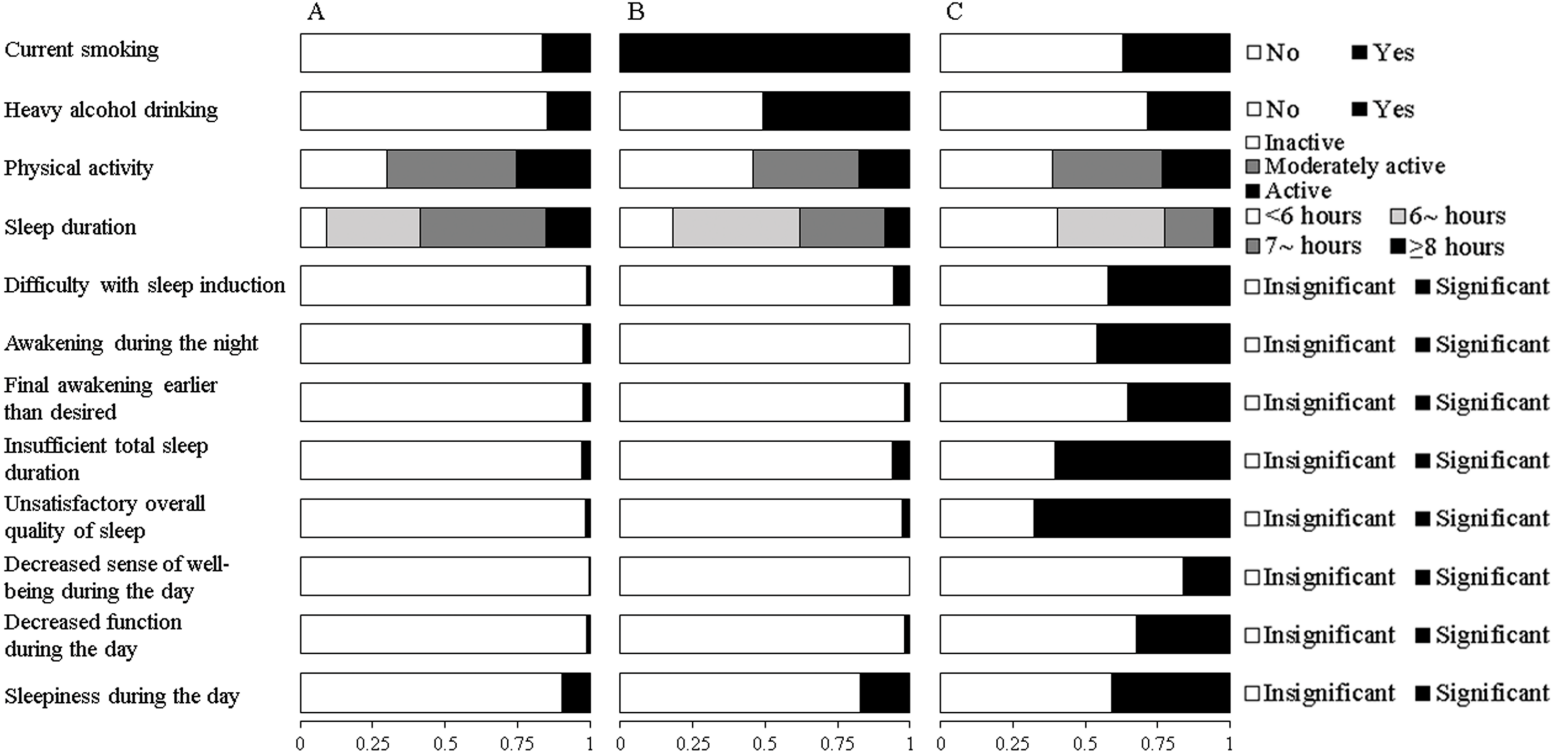
Estimated response probabilities for health risk behaviors within the three classes among males. (A) Healthy lifestyle (Class 1, 62.9%). (B) Cumulate harmful habits (Class 2, 27.1%). (C) Poor sleep and risky habits (Class 3, 10.0%). The bars represent the response probabilities (0 to 1.0) for each item in respective class.

**Figure 2:**
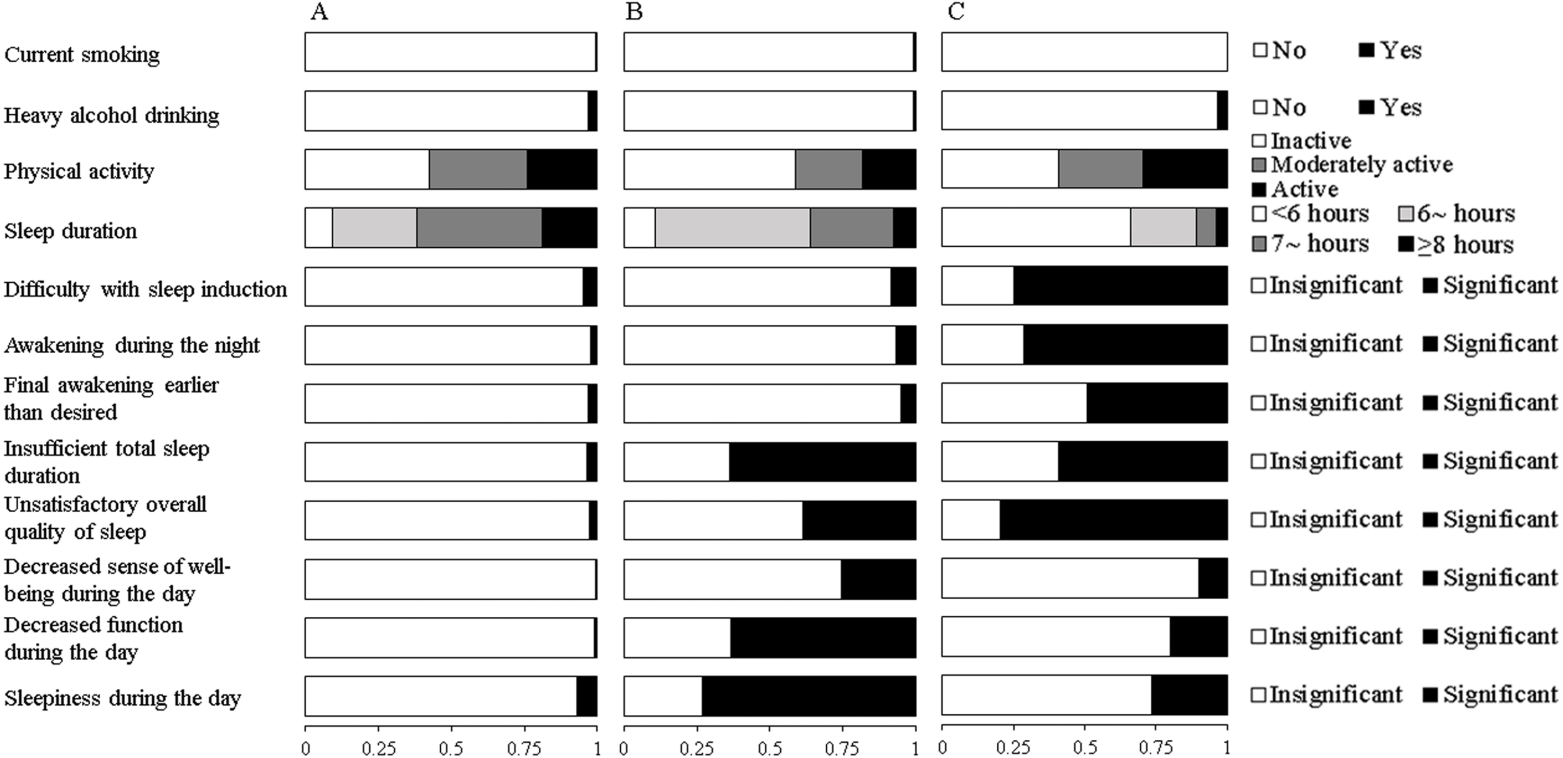
Estimated response probabilities for health risk behaviors within the three classes among females. (A) Healthy lifestyle (Class 1, 83.0%). (B) Inactive, daytime dysfunction (Class 2, 5.7%). (C) Poor sleep habits (Class 3, 11.3%). The bars represent the response probabilities (0 to 1.0) for each item in respective class.

Fit indices for LCA models with 2-5 classes within each gender were shown in Table 2. Based on the LMR likelihood ratio test, the best fitting model was a 3-class solution among males and a 4-class solution among females. Yet, we did not find any new unique behavioral pattern in the 4-class solution among females after a comparison of model interpretability. Therefore, the three-class latent model was selected as the final solution for both genders. The average posterior probabilities for most likely latent class were high for all classes (Male: Class 1 87.0%, Class 2 80.4%, Class 3 88.8%; Female: Class 1 92.5%, Class 2 97.1%, Class 3 83.5%), suggesting a good classification feature. Differential profiles of each class were identified by the estimated item response probabilities of HRBs and presented in Fig. 1 for males and Fig. 2 for females.

**Table 2:** Goodness of fit statistics for gender-specific LCA models with different number of latent classes.

	Males				Females			
	2 class	3 class	4 class	5 class	2 class	3 class	4 class	5 class
AIC	38,015.0	37,804.0	37,622.8	37,500.0	19,725.4	19,560.4	19,399.6	19,347.1
BIC	38,206.8	38,094.8	38,012.6	37,988.7	19,901.2	19,826.8	19,756.8	19,795.0
ABIC	38,108.3	37,945.4	37,812.4	37,737.7	19,802.7	19,677.5	19,556.6	19,544.0
Entropy	0.884	0.648	0.686	0.882	0.888	0.721	0.851	0.831
LMR	<0.001	0.026	0.113	0.143	<0.001	0.075	<0.001	0.093

**Notes.**

LCA latent class analysis AIC Akaike information criterion BIC Bayesian information criterion ABIC adjusted Bayesian information criterion LMR Lo-Mendell-Rubin likelihood ratio test

Among males, the first and largest class (62.9%) was characterized by the lowest probabilities of traditional HRBs, sleeping <7 h and self-reported symptoms of insomnia. This class was labeled as “healthy lifestyle”, although their probability of being physically active was low (25.6%). The second class (27.1%), labeled “cumulate harmful habits”, was characterized by the highest likelihood for smoking, heavy drinking and physical inactivity, as well as moderate probability of sleeping <7 h. The third class (“poor sleep and risky habits”, 10.0%), had the highest probabilities of sleeping <7 h and self-reported symptoms of insomnia, coupled with moderate probabilities of smoking and heavy drinking.

Among females, the first class accounted for 83.0% of the sample and was characterized by low probabilities of all HRBs except low physical activity. Similar to that in males, this class was labeled “healthy lifestyle”. The second class (5.7%) might be labeled “inactive, daytime dysfunction”. Participants in this class were likely to be physically inactive and reported the highest probabilities of abnormal daytime functioning and sleepiness. The third class comprising 11.3% of females, exhibited the highest probabilities of short sleep duration and self-reported nocturnal symptoms of insomnia. This class was labeled “poor sleep habits”.

### Associations of latent class with diabetes

Means and percentages of demographics and metabolic indices in different classes, stratified by gender, were summarized in Table 3. In both genders, the three classes differed significantly in terms of age, systolic BP, FPG, and the prevalence of diabetes. Participants in Class 3 had the highest average age, systolic BP, FPG, and proportion of diabetes. In addition, men in Class 3 also had higher diastolic BP, when comparing to men in the other two classes. Furthermore, men in Class 2 (“cumulate harmful habits”) showed the highest BMI level compared to men in other classes.

**Table 3:** Demographic and clinical outcomes of study participants within each latent class by gender.

	Males			*F*/*χ*^2^	*P*	Females			*F*/*χ*^2^	*P*
	Class 1 (*n* = 2,259)	Class 2 (*n* = 974)	Class 3 (*n* = 360)			Class 1 (*n* = 1,778)	Class 2 (*n* = 122)	Class 3 (*n* = 241)		
Age (years), Mean ± SD	47.2 ± 9.8	47.6 ± 8.4	49.6 ± 10.5 ^a^^,^^b^	10.126	<0.001	47.6 ± 9.6	46.7 ± 10.5	54.8 ± 10.3 ^a^^,^^b^	59.352	<0.001
Collage and above education, *n* (%)	1373 (60.8)	585 (60.1)	206 (57.2)	1.656	0.437	1,068 (60.1)	81 (66.4)	107 (44.4) ^a^^,^^b^	24.676	<0.001
Family history of diabetes, *n* (%)	410 (18.1)	187 (19.2)	79 (21.9)	3.057	0.217	454 (25.5)	28 (23.0)	76 (31.5)	4.616	0.099
Body mass index (SD), kg/m^2^	24.3 ± 2.8	24.6 ± 2.9 ^a^	24.1 ± 2.8^b^	6.814	0.001	22.6 ± 2.8	22.5 ± 3.1	23.0 ± 3.3	2.259	0.105
SBP (mmHg), Mean ± SD	131.1 ± 16.2	129.8 ± 15.2	134.0 ± 18.4 ^a^^,^^b^	8.939	<0.001	126.4 ± 19.0	123.6 ± 18.5	133.7 ± 22.9 ^a^^,^^b^	16.945	<0.001
DBP (mmHg), Mean ± SD	78.7 ± 10.8	79.0 ± 10.4	80.8 ± 12.8 ^a^^,^^b^	5.629	0.004	74.7 ± 10.6	72.9 ± 10.2	75.4 ± 11.3	2.206	0.110
FPG (mmol/L), Mean ± SD	5.1 ± 1.1	5.2 ± 1.4	5.5 ± 2.0 ^a^^,^^b^	17.762	<0.001	5.0 ± 1.0	5.1 ± 1.0	5.4 ± 1.7 ^a^^,^^b^	13.819	<0.001
Diabetes, *n* (%)	113 (5.0)	89 (9.1) ^a^	49 (13.6) ^a^^,^^b^	44.939	<0.001	65 (3.7)	9 (7.4)	29 (12.0) ^a^	34.386	<0.001

**Notes.**

SD standard deviation SBP systolic blood pressure DBP diastolic blood pressure FPG fasting plasma glucose

a Significantly different from Class 1 (p for the difference <  0.05).

b Significantly different from Class 2 (p for the difference <  0.05).

Latent class membership was significantly associated with the risk for diabetes (Table 4). Compared with the “healthy lifestyle” class, all other latent classes were more likely to have diabetes for both men and women after adjustment for covariates. There was no significant difference in the risk of diabetes between the Class 2 and Class 3 for both genders. In sensitivity analyses excluding patients with self-reported cardiometabolic diseases, the associations between behavioral latent class and diabetes risk remained statistically significant.

**Table 4:** Odds ratios and 95% confidence intervals for the relationships between specific lifestyle classes with risk of diabetes.

	Main analyses				Sensitivity analyses^a^			
	Crude OR (95% CI)	*P*	Adjusted OR (95% CI)^b^	*P*	Crude OR (95% CI)	*P*	Adjusted OR (95% CI)^b^	*P*
Males								
(Reference: Healthy lifestyle)								
Cumulate harmful habits	1.91 (1.43–2.55)	<0.001	2.09 (1.54–2.83)	<0.001	2.60 (1.79–3.76)	<0.001	2.69 (1.83–3.94)	<0.001
Poor sleep and risky habits	2.99 (2.10–4.27)	<0.001	2.62 (1.79–3.82)	<0.001	3.87 (2.44–6.14)	<0.001	3.29 (2.02–5.34)	<0.001
(Reference: Cumulate harmful habits)								
Poor sleep and risky habits	1.57 (1.08–2.27)	0.018	1.25 (0.84–1.87)	0.271	1.49 (0.94–2.36)	0.088	1.22 (0.75–2.00)	0.421
Females								
(Reference: Healthy lifestyle)								
Inactive, daytime dysfunction	2.10 (1.02–4.32)	0.044	2.41 (1.06–5.45)	0.035	2.15 (0.74–6.25)	0.160	3.14 (1.00–9.84)	0.050
Poor sleep habits	3.61 (2.28–5.71)	<0.001	1.92 (1.16–3.27)	0.011	3.93 (1.96–7.88)	<0.001	2.48 (1.18–5.22)	0.017
(Reference: Inactive, daytime dysfunction)								
Poor sleep habits	1.72 (0.79–3.75)	0.175	0.81 (0.33–1.98)	0.645	1.83 (0.57–5.83)	0.307	0.79 (0.23–2.76)	0.711

**Notes.**

OR odds ratio CI confidence interval

a Sensitivity analyses by excluding patients with self-reported history of cardiometabolic diseases (including hypertension, coronary heart disease, stroke, and diabetes).

b Estimates were adjusted for age, education level, family history of diabetes, body mass index, and systolic blood pressure.

## Discussion

### Main findings of this study

Our findings indicated that there is a meaningful patterning of HRBs among adults aged 35–75 years, suggesting an important linkage between smoking, alcohol use, physical activity, and sleep duration and quality. By using LCA, we identified three distinct behavioral patterns for both men and women. Although some patterns appeared to be similar in both genders (such as healthy lifestyle), the class membership and characteristics were quite different between males and females. Also, gender-specific behavioral profiles were found, including “cumulate harmful habits” and “poor sleep and risky habits” among men and “inactive, daytime dysfunction” and “poor sleep habits” among women. In addition, our analysis revealed that high-risk behavioral patterns (subjects who were classified into Class 2 and Class 3) were associated with a greater likelihood of diabetes in the total study population, regardless of gender. To our best knowledge, this is the first study to investigate the lifestyle clustering patterns with a combination of established and emerging behavioral risk factors and their associations with diabetes in Southern Chinese population.

### Clustering of health risk behaviors

The vast majority of participants in our sample, particularly females, adhered to a healthy lifestyle. This is consistent with previous literatures from other countries. In a study of German adults (Atzendorf et al., 2018), 58.5% of the population belonged to a subgroup with near-zero probabilities for most of the lifestyle risk factors. Another study conducted in 27 European countries showed that 57.65% of adults were classified as healthy with respect to smoking, alcohol and fresh fruit consumption, physical activity, and dental check-ups (Kino, Bernabe & Sabbah, 2017). The results reinforce the idea that health behaviors tend to cluster at the upper and lower ends of the spectrum (Noble et al., 2015). However, both current and previous findings revealed that even individuals in the “healthy” class exhibited low physical activity (Atzendorf et al., 2018; De Mello et al., 2019). It reflects the difficulties for general adults in self-regulatory behaviors like physical activity, and highlights the need to increase health services concerning health-promoting behaviors.

Our results indicated that smoking, heavy alcohol drinking, and physical inactivity co-occur among males. Specifically, individuals in the “cumulate harmful habits” group reported the highest prevalence of certain behaviors concurrently. This is coherent with the previous literature (Atzendorf et al., 2018; Kino, Bernabe & Sabbah, 2017; Noble et al., 2015), suggesting that there may be a common underlying cause of HRBs. Additionally, we also observed an association between sleep problems and risky behaviors. Among the “cumulate harmful habits” group, men engaged in smoking, heavy drinking, and/or physical inactivity tended to have a relatively high prevalence of short sleep duration; and that within the “poor sleep and risky habits” group, those with severe insomnia symptoms would likely experience high rates of smoking and heavy drinking. Such relationships were noted in several recent studies (Haario et al., 2013; Rognmo et al., 2019), which found a bidirectional association between risky behaviors and sleep problems. Though the underlying mechanism for such associations remains poorly understood, it implies the practical need for simultaneously treating multiple HRBs in an integrated approach.

Consistent with previous literature (Merikanto et al., 2012) , we found short sleep duration is highly comorbid with insomnia symptoms. In the “poor sleep habits”/“poor sleep and risky habits” class, people exhibited an overall deterioration in sleep quantity and quality. Yet some recent studies demonstrated that sleep disturbance is highly heterogeneous and may present multiple phenotypes (Benjamins et al., 2017; Gadie et al., 2017). This is due in part to the heterogeneity of the sleep measures and analytical methods between different studies. Further research should investigate the variability and subtypes of sleep disturbances, as well as potential interventions to prevent these disorders.

Furthermore, we observed a likelihood that inactivity co-occurs with perceived insufficient total sleep duration, daytime dysfunction and sleepiness among females. This finding is in agreement with data from previous work, where physically active individuals were less likely to report sleep disturbances and daytime tiredness (McClain et al., 2014; Tu et al., 2012). Nevertheless, the relationship between physical activity and sleep remains unclear. Several studies found more physical activity has no or even a negative relation to sleep quality (Kakinami et al., 2017; Taylor et al., 2019). Further research is necessary in large populations to answer these questions.

### Association between behavioral clusters and diabetes

Findings from this work replicate prior studies which have likewise demonstrated associations between clustering of HRBs and diabetes (Lv et al., 2017; Shan et al., 2018). Compared with the “healthy lifestyle” group, all the high-risk lifestyle subgroups were substantially associated with an increased risk of diabetes. Moreover, the magnitudes of these associations did not differ between two high-risk subgroups within each gender, although their behavioral profiles are not completely consistent. Specifically, the odds ratios of diabetes were not statistically significant between the “cumulate harmful habits” and “poor sleep and risky habits” groups for males, as well as those between the “inactive, daytime dysfunction” and “poor sleep habits” for females. These results were robust in sensitivity analysis by excluding patients with self-reported cardiometabolic diseases. It is implied that different types of behavioral combination may lead to a similar risk of health outcome, and that future researches and intervention strategies should take into consideration the specific behavioral pattern of each subgroup. In addition, considering that there was gender disparity in the occurrence of HRBs, efforts to formulate gender-specific interventions to promote health behaviors is necessary. To reduce the risk of diabetes, interventions targeting harmful habits (e.g., smoking and heavy drinking) and overall sleep disturbance would be essential for males, while those for females should focus on modifying daytime activities and nocturnal insomnia.

### Limitations

There are several limitations in our research. First, this study was a retrospective analysis using routine health examination data from the previous years, which was not collected for our study purpose. Hence, we were limited by the measures available from the health records and missed information on other important HRBs (e.g., dietary behaviors) and socioeconomic covariates (e.g., income). Because information on glucose tolerance was unavailable, potential exists for underestimation of diabetes prevalence and its association with lifestyle clusters. Additionally, we assumed that behavioral patterns in our sample have remained stable over the past few years, which might be very bold and radical. Yet there is evidence that health behavior profiles are quite stable over time among adults (Burgard et al., 2020). Second, the behavioral measurements were based on self-reported data, which is potentially subject to social desirability bias (Crutzen & Goritz, 2010). Therefore, the proportions of individuals classified into high-risk groups and their diabetes risks are likely to be underestimated. Third, the study sample was restricted to participants who underwent health examinations in a single center located in a large urban setting, which was not representative of the general population; thus, our findings should be interpreted cautiously if they can be generalized to other Chinese populations. Finally, the cross-sectional study design precluded the determination of causality. For example, it is not known whether multiple risk behaviors occurred simultaneously or whether the adoption of one risk behavior leads to an increased risk of other risk behaviors. Future work using longitudinal designs with regard to this topic is needed.

## Conclusions

The LCA analysis found different patterns of HRBs in a large sample of Southern Chinese men and women. These findings suggest that risk behaviors tend to cluster and that such behavioral clustering can provide useful information for identifying vulnerable subgroups. Furthermore, the observed high-risk behavioral patterns characterized by engaging in multiple HRBs are associated with an increased risk of diabetes. Intervention strategies considering the clustering patterns of multi-behavioral risk factors should be implemented to prevent the growing epidemic of diabetes.

##  Supplemental Information

10.7717/peerj.9025/supp-1 Supplemental Information 1 Latent analysis of HRBs clustering Items of health risk behaviors and covariates.

## Additional Information and Declarations

### Data Availability

The following information was supplied regarding data availability: The raw data are available in a Supplementary File.
